# Editorial: Cardiovascular adaptation to extreme environment, volume II

**DOI:** 10.3389/fcvm.2023.1296422

**Published:** 2023-10-23

**Authors:** Alessandro Pingitore, Francesca Mastorci, Marco Laurino, Claudio Marabotti, Cristina Vassalle

**Affiliations:** ^1^ Clinical Physiology Institute, CNR, Pisa, Italy; ^2^ Fondazione CNR-Regione Toscana G. Monasterio, Pisa, Italy

**Keywords:** cardiovascular, exercise, extreme, adaptation, prevention

**Editorial on the Research Topic** Cardiovascular adaptation to extreme environment, volume II

Extreme physiological conditions can be environmental, such as hypobaric (space or altitude) and hyperbaric (diving) ones, or situational, like strenuous exercise. In all these conditions, the human body needs to respond acutely in order to maintain its own homeostasis and, if the same condition repeats continuously, to adapt with deep changes in its own systemic physiology. This is important to avoid allostatic load that may induce a deficiency of the systems designed to respond to stress, ultimately resulting in tissue damage.

The articles published in the second volume of the “Cardiovascular Adaptation to Extreme Environment, Volume II” deal with different aspects in this field, three conducted on humans (one on hyperbaric diving conditions and two situational conditions on elite athletes), one evaluating *ex-vivo* hypobaric spaceflight effects on cardiomyocytes, and one experiment aiming to evaluate the effects of hypoxic adaptation on gut microbiota in rodents.

Scuba diving is a widespread and popular sport, with an increasing number of new practitioners every year, often performed as a recreational activity with possible adverse effects for health. This is because sport in extreme environmental conditions, such as scuba diving, may affect body homeostasis. Njire Braticevic et al. studied the impact of recreational scuba diving (practiced once/week, at depths of 20–30 m, lasting 30 min, between 9 and 10 a.m.) on the neurohormonal system and the muscle-brain endocrine loop, by measuring levels of different biomarkers (ACTH, cortisol, TSH, free thyroxine, prolactin, total testosterone, GH, and insulin-like growth factor 1, irisin, BDNF, S100B, neuron-specific enolase, and glial fibrillary acidic protein) (Njire Braticevic et al.). Their results evidenced changes in myokine and hormone levels in the muscle-brain endocrine loop response, serving as a basis to further explore the molecular events underlying functional adaptation to the extreme environment in which diving occurs.

Yang et al. focused on diastolic cardiac hemodynamics of the left ventricle in elite female ice hockey athletes (a high intensity sport), which resulted in a prolonged diastolic intraventricular pressure difference rather than an enhanced peak amplitude. It was also found that P1P4 prolonged with an increase in the training years, reflecting a time adaptation following long-term training (Yang et al.). Results obtained in this study may help to further understand the hemodynamic mechanism of cardiac adaptation to the training load and duration.

It is important to know which determinants may improve performance in elite athletes. In this context, Knechtle et al. reported on a case aiming to describe the biophysical characteristics of the first ultra-cyclist in the world to break the 1,000 km barrier in 24-h non-stop road cycling, and to compare the performances of the same ultra-cyclist in achieving two world records in 24 h cycling, which may improve technical advancement (e.g., technical specifications to improve aerodynamic and friction, adoption of a stream lined position, and monitoring heart rate variations to regulate the physical and physiological demand) (Knechtle et al.).

The study of pathophysiology in the spaceflight environment offers an extraordinary possibility to study how these extreme conditions affect living systems (1). Bisserier et al. evidenced how spaceflight may alter the transcriptome profile of small extracellular vesicles (sEVs, such as exosomes) (Bisserier et al.). In fact, astronaut-derived sEVs may epigenetically modulate the expression of vitamin D receptors in human adult cardiomyocytes by inducing the activation of the PRC2 polycomb repressive complex 2 and increasing H3K27me3 levels.

Gut microbiota and its functional parameters (e.g., lipoprotein particle composition, fatty acid saturation, and carbohydrate and sugar derivative metabolism) could predict the risk of cardiovascular disease and its consequences (2). Some experimental data indicated that modulation of specific microbial metabolites (e.g., inhibition of TMA) may reduce intermittent hypoxia-induced pulmonary artery atherosclerosis in an obstructive sleep apnea-associated mouse model (3). In this volume, Lin et al. evidenced changes in the gut microbiota of Gansu zokor (a common Chinese rodent), which mediate carbohydrate metabolic pattern under hypoxia, contributing to optimal adaptation to hypoxic environments, typical for this animal's environment (Lin et al.). These findings suggest that new therapeutic strategies based on the modulation of the gut microbial composition might affect host cardiovascular phenotype and improve human cardiovascular health.

Taken together, these studies demonstrate that knowledge of chronic adaptive capacity or acute responses to extreme conditions, whether environmental, such as spaceflight, or physical, such as strenuous physical activity, can serve as a physiological model for the study of pathophysiological conditions. One could paradoxically argue that the acute physiological responses to strenuous exercise can be superimposed on that of acute pathology, and this is because our organism tends in both cases toward survival and self-protection, in line with the principles of homeostasis. The definition of homeostasis assumes that protective mechanisms at the cellular or organ level are driven by moderate and repeated stress stimuli which results in beneficial biological effects. An athlete's training, for example, should have this purpose and that is why it must be progressive, allowing adaptation to increasingly intense workloads. The difference is that intense exercise happens within a limited timeframe so that the systems predisposed to our body's protective response are activated and shut down with the completion of exercise. In pathology, these systems continue to be activated over time and this leads to their ineffective protective response but also to a potential toxic effect that could facilitate disease progression. This continuous activation results in allostatic loading, which in the athlete, for example, could be identified with overtraining. A typical example is neuroendocrine activation in heart failure, which can be considered a scenario of altered systemic homeostasis, in which continuous activation of the sympathetic activation may predispose an individual to allostatic load, which is defined by McEwan as “the cumulative strain on the body produced by repeated ups and downs of physiologic response, as well as by the elevated activity of physiologic systems under challenge and the changes in metabolism and the impact of wear and tear on a number of organs and tissue, which “can predispose the organism to disease”. In proof of this, beta blocker therapy is one of the pharmacological mainstays in heart failure. Therefore, we could say that allostatic load is the price the body pays for being continuously forced to adapt to adverse physiological or pathophysiological conditions. It will be interesting in the future to study the similarities and differences that exist at the molecular and cellular levels between the responses to an acute and strenuous stimulus due to intense physical exertion and those that lead to the onset and development of the various chronic-degenerative diseases (cardiovascular and neurodegenerative disease and cancer) in order to develop targeted preventive and beneficial strategies.
